# Comparative pharmacokinetics of polymyxin B in critically ill elderly patients with extensively drug-resistant gram-negative bacteria infections

**DOI:** 10.3389/fphar.2024.1347130

**Published:** 2024-02-01

**Authors:** Juan Zeng, Bing Leng, Xiaoyan Guan, Shuangyan Jiang, Maoyu Xie, Wenying Zhu, Yue Tang, Lin Zhang, Jing Sha, Tengfei Wang, Min Ding, Nan Guo, Jinjiao Jiang

**Affiliations:** ^1^ Department of Critical Care Medicine, Shandong Provincial Hospital Affiliated to Shandong First Medical University, Jinan, Shandong, China; ^2^ Department of Pharmacy, Shandong Provincial Hospital Affiliated to Shandong First Medical University, Jinan, Shandong, China; ^3^ Department of Emergency, Shandong Provincial Hospital Affiliated to Shandong First Medical University, Jinan, Shandong, China

**Keywords:** polymyxin B, pharmacokinetics, elderly, critical care, total body weight, renal function

## Abstract

**Introduction:** Elderly patients are more prone to develop acute kidney injury during infections and polymyxin B (PMB)-associated nephrotoxicity than young patients. The differential response to PMB between the elderly and young critically ill patients is unknown. We aimed to assess PMB exposure in elderly patients compared with young critically ill patients, and to determine the covariates of PMB pharmacokinetics in critically ill patients.

**Methods:** Seventeen elderly patients (age ≥ 65 years) and six young critically ill patients (age < 65 years) were enrolled. Six to eight blood samples were collected during the 12 h intervals after at least six doses of intravenous PMB in each patient. PMB plasma concentrations were quantified by high-performance liquid chromatography-tandem mass spectrometry. The primary outcome was PMB exposure as assessed by the area under the concentration-time curve over 24 h at steady state (AUC_ss, 0–24 h_).

**Results and Discussion:** The elderly group had lower total body weight (TBW) and higher Charlson comorbidity scores than young group. Neither AUC_ss, 0–24 h_ nor normalized AUC_ss, 0–24 h_ (adjusting AUC for the daily dose in mg/kg of TBW) was significantly different between the elderly group and young group. The half-life time was longer in the elderly patients than in young patients (11.21 vs 6.56 h respectively, *p* = 0.003). Age and TBW were the covariates of half-life time (*r* = 0.415, *p* = 0.049 and *r* = −0.489, *p* = 0.018, respectively). TBW was the covariate of clearance (*r* = 0.527, *p* = 0.010) and AUC_ss, 0–24 h_ (*r* = −0.414, *p* = 0.049). Patients with AUC_ss, 0–24 h_ ≥ 100 mg·h/L had higher baseline serum creatinine levels and lower TBW than patients with AUC_ss, 0–24 h_ < 50 mg·h/L or patients with AUC_ss, 0–24 h_ 50–100 mg·h/L. The PMB exposures were comparable in elderly and young critically ill patients. High baseline serum creatinine levels and low TBW was associated with PMB overdose.

**Trial registration:** ChiCTR2300073896 retrospectively registered on 25 July 2023.

## 1 Introduction

The emergence of extensively drug-resistant Gram-negative bacteria (XDR-GNB) has become a major global health challenge because of the rapid resistance transmission, increasing treatment difficulties, and association with high morbidity and mortality rates (Borer et al., 2009; Cassini et al., 2019). Polymyxins including colistin and polymyxin B (PMB) have excellent antibacterial activity against the majority of XDR-GNB as reported by recent surveillance surveys (Diekema et al., 2019; Xi et al., 2022) and have been revived as the last-resort therapeutic opinion for XDR-GNB infections (Katip et al., 2020; Katip et al., 2021; Liu et al., 2023). PMB is an active drug without requiring conversion to colistin after its administration, whereas colistin must be administered as an inactive prodrug, colistin methanesulfonate, to then be converted to colistin *in vivo*, which could be slow and incomplete. Therefore, PMB may be the preferred polymyxin for XDR-GNB infections. However, PMB has a narrow therapeutic window and high occurrence of nephrotoxicity, the most commonly reported adverse effect of PMB (Rigatto et al., 2016; Tsuji et al., 2019). Because the plasma concentration of PMB is closely related to its antibacterial efficacy and nephrotoxic effect, the area under the concentration-time curve over 24 h at steady state (AUC_ss, 0–24 h_) of 50–100 mg·h/L was recommended for the therapeutic drug concentration, which is equivalent to an average plasma concentration at steady state (C_ss, avg_) of 2–4 mg/L (Tsuji et al., 2019). If the AUC_ss, 0–24 h_ of PMB is more than 100 mg·h/L, the incidence and severity of nephrotoxicity increase significantly (Wang et al., 2021a; Yang et al., 2022).

PMB has been reported to accumulate in large quantities in the kidney, much more than in other organs, such as the heart, lungs, and brain. In the kidney, PMB accumulates mainly in the proximal tubule after intravenous injection, as demonstrated by animal model studies (Manchandani et al., 2015; Yun et al., 2015). Considering that PMB is poorly recovered in urine (less than 1%) (Zavascki et al., 2008; Yun et al., 2015), it may be filtered from the glomerulus and reabsorbed in the proximal tubule. Hence, the accumulation of PMB in the renal proximal tubule has been proposed as the possible main mechanism of PMB-related nephrotoxicity (Yun et al., 2015; Manchandani et al., 2017).

The elderly population, as a special group, has different characteristics from the young and middle-aged population in terms of disease progression and drug metabolism, distribution, and clearance. Elderly patients are more prone to develop multiple organ dysfunctions when infected than young patients. A large epidemiological survey has demonstrated that the incidence of sepsis increases with age, with the incidence in patients over 85 years being 100 times higher than in children (Angus et al., 2001), and nearly 65% of septic patients are elderly (Martin et al., 2006). Additionally, advanced age has been shown to be an independent risk factor for sepsis-associated acute kidney injury (AKI) (Poston et al., 2019). Meanwhile, the physiological changes in elderly subjects, such as the reduced glomerular filtration rate and lower tubular reabsorption capacity, may result in increased PMB exposure to the renal tubular cells that increase the occurrence of nephrotoxicity (Manchandani et al., 2017). Several studies have confirmed that older patients have significantly increased risk of polymyxins-related nephrotoxicity than younger patients (Wagenlehner et al., 2021; Yang et al., 2022).

The current international guideline recommends that the dose of PMB should be calculated based on the patient’s weight, irrespective of age (Tsuji et al., 2019). Several population pharmacokinetics (PK) studies have reported that PMB clearance is independent of age in critically ill patients or patients with cystic fibrosis (Sandri et al., 2013; Avedissian et al., 2018; Manchandani et al., 2018; Wang et al., 2020), whereas one study has found that age is significantly related with the volume of distribution of PMB (Liang et al., 2023). Whether elderly patients have higher PMB exposure than young critically ill patients and different clearance and volume of distribution from young patients given the standard dose has remained unknown until now. Therefore, we conducted this prospective observational clinical study aimed to compare PMB exposure in elderly patients and young critically ill patients and to explore potential covariates of PMB PK in critically ill patients.

## 2 Materials and methods

### 2.1 Patient selection

All adult patients admitted to the participating intensive care unit (ICU) in a teaching hospital between December 2021 and July 2023 were consecutively screened for entry into this prospective observational study. The ICU served a mixed population of medical, surgical, trauma, and neurologic patients. All critically ill patients (≥18 years) were eligible if they received intravenous PMB for more than 3 days (sulfate; PMB injection, Shanghai First Biochemical Pharmaceutical Co., Ltd., China) for the treatment of documented XDR-GNB infections. Exclusion criteria were 1) patients with anuria or who received blood purification therapy or extracorporeal membrane oxygenation during PMB treatment; 2) pregnant women. Informed written consent was obtained from each patient or a legal representative of the family. This study protocol was approved by the human ethics committees in Shandong Provincial Hospital Affiliated to Shandong First Medical University (protocol SWYX: No. 2021-356) and was carried out in accordance with the Declaration of Helsinki (2000) of the World Medical Association.

Patients were enrolled on the first day of intravenous PMB administration. This study continued for 28 days or until discharge from the hospital or death. According to the patient’s age, the enrolled patients were divided into two groups: the elderly group (≥65 years) and the young group (<65 years).

### 2.2 Polymyxin B dosing regimens and plasma concentration determination

PMB was administrated intravenously with a loading dose of 2.0–2.5 mg/kg and maintenance doses of 1.25–1.5 mg/kg every 12 h, with at least 1 h infusion time. Calculation of both the loading dose and maintenance dose was based on the total body weight (TBW) of the patient. No dose adjustment was performed in patients with renal insufficiency and renal replacement therapy. Concurrent prescription of other potential nephrotoxic drugs, such as vancomycin, aminoglycoside, etc., was avoided as much as possible at any time during PMB treatment in our study. The initiation of PMB treatment as well as the concomitant antibiotics and their duration were at the discretion of the ICU physicians.

For steady-state PK assessment, blood samples were obtained after at least six doses of PMB. In each patient, six to eight blood samples (3 mL) were collected immediately 10 min before the infusion and 30 min and 1, 2, 3, 6, 8, and 12 h after the beginning of infusion in ethylenediaminetetraacetic acid anticoagulant tubes. Blood samples were immediately centrifuged at 3500 g for 10 min, and the plasma samples were collected and stored at −80°C until analysis. The PMB plasma concentrations were quantified by measuring both polymyxin B1 and B2 using validated high-performance liquid chromatography-tandem mass spectrometry. Analysis of independently prepared quality control samples showed good precision (coefficients of variation ≤6.83% for polymyxin B1 and ≤10.67% for polymyxin B2) and accuracy (measured concentrations from target concentrations ≤6.87% for polymyxin B1 and ≤4.54% for polymyxin B2).

### 2.3 Microbiology

Samples from XDR-GNB infection sites were sent to the microbiology laboratory for culture of XDR-GNBs. Susceptibility to carbapenems of the causative XDR-GNB was determined according to the 2019 European Committee on Antimicrobial Susceptibility Testing breakpoint. Enterobacteriaceae with minimum inhibitory concentration (MIC) ≥4 mg/L, and *Pseudomonas aeruginosa* and *Acinetobacter baumannii* with MIC ≥8 mg/L were considered as carbapenem resistance. The above bacteria strains with MIC ≤2 mg/L were considered PMB sensitive and MIC ≥4 mg/L were considered PMB resistant. Antimicrobial susceptibility testing was determined by the VITEK 2 compact system with GN13 cards (bioMérieux, France).

### 2.4 Data collection

The following clinical data were collected: age, gender, TBW, admission category (medical, elective surgical, or emergency surgical), Charlson comorbidity index score at ICU admission, length of ICU stay before enrollment, Sequential Organ Failure Assessment (SOFA) (range 0–24, with higher scores indicating worse outcome) and Acute Physiology and Chronic Health Evaluation II (APACHE II) scores (range 0–71, with higher scores indicating more severe illness) within 24 h after enrollment, creatinine clearance (CrCl) (calculated using the Cockcroft-Gault equation) at baseline, serum creatinine levels, sites of primary infection, and causative XDR-GNB strains. We also monitored prospectively the data about PMB treatment (loading dose, total daily maintenance dose, and concomitant antibiotics).

### 2.5 Statistical analysis

The primary outcome was steady-state PMB exposure as reflected by AUC_ss, 0–24 h_. Non-compartmental PK analysis was conducted using Phoenix WinNonlin software 8.3 (Certara, United States). The clinical variables were summarized as means with standard deviation for approximately normally distributed continuous variables or medians with interquartile range for non-normally distributed continuous variables and as frequencies and percentages for categorical variables. The normality of all data sets was determined using the Kolmogorov-Smirnov test. Categorical variables were analyzed by the chi-square or Fisher exact test, while continuous variables were analyzed by the two-tailed non-paired Student’s t-test or Mann-Whitney U test. The following parameters were examined as potential covariates affecting PMB PK (AUC_ss, 0–24 h_, C_ss, avg_, clearance, volume of distribution, and half-life time): age, TBW, SOFA score, total serum protein, albumin, serum creatinine levels, CrCl, fibrinogen, international normalized ratio (INR), and total bilirubin. All the analyses were performed with the use of the SPSS for Windows statistical program (version 22.0; IBM Corp., Armonk, NY). Statistical significance was set at *p* < 0.05, and highly significant values had a significance of *p* < 0.01.

## 3 Results

### 3.1 Characteristics of patients at enrollment and clinical infection

**FIGURE 1:**
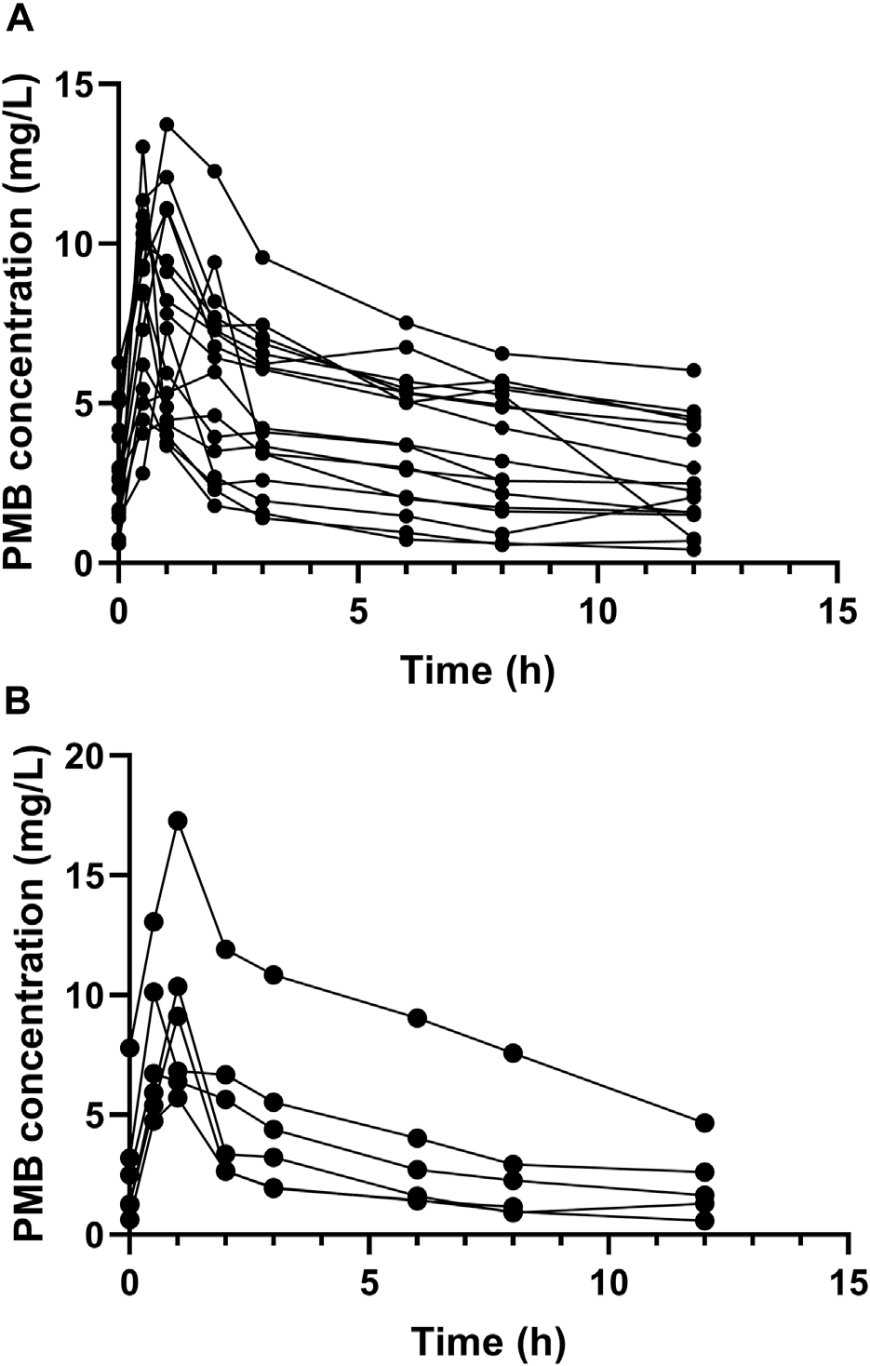
Plasma concentration-time profile of polymyxin B at steady state. Circles represent multiple time point sampling. **(A)** the elderly patients; **(B)** the young patients.

A total of 23 critically ill patients who received PMB for XDR-GNB infections were enrolled in this study, of whom 17 were elderly patients and the other 6 were young patients. A total of 181 blood samples were collected (Figure 1). The elderly patients had lower TBW and higher Charlson Comorbidity Index than the young patients. There were more ICU admissions due to medical diseases and respiratory failure at enrollment in the elderly patients than in the young patients. No significant differences were observed between the elderly and the young patients in terms of APACHE II scores, SOFA scores at enrollment, sex distribution, ICU stay before enrollment, the estimated CrCl, the serum creatinine levels, and the proportion of patients with CrCl <80 mL/min at baseline as listed in Table 1.

**TABLE 1:** Characteristics of patients at baseline and infection.

Characteristics	All patients (*n* = 23)	Elderly group (*n* = 17)	Young group (*n* = 6)	*p*-value
Age (years)	70.2 ± 13.7	77.1 ± 7.8	50.8 ± 3.9	<0.001
Sex (Male) [no. (%)]	16 (69.6)	10 (58.0)	6 (100)	0.124
TBW (Kg)	67.4 ± 8.5	64.4 ± 7.7	75.8 ± 3.8	0.002
Charlson Comorbidity Index scores	8 (4–9)	8 (7–9)	4 (3–5)	0.025
APACHE II scores	29.4 ± 7.5	30.2 ± 7.4	27.2 ± 7.9	0.401
SOFA scores	9.6 ± 4.5	9.4 ± 4.1	10.3 ± 5.9	0.656
ICU stay before enrollment (days)	11 (4–28)	10 (4–19)	12.5 (4–28)	0.916
Admission group (no. (%))				<0.001
Medical	18 (78.3)	17 (100)	1 (16.7)	
Elective surgical	2 (8.7)	0 (0)	2 (33.3)	
Emergency surgical	3 (13.0)	0 (0)	3 (50.0)	
Main reason for ICU enrollment [no. (%)]				0.036
Sepsis	6 (26.1)	3 (17.6)	3 (50.0)	
Respiratory failure	8 (34.8)	8 (47.1)	0 (0)	
Others^a^	9 (39.1)	6 (35.3)	3 (50.0)	
CrCl (mL/min)	105.4 ± 54.0	102.0 ± 54.2	114.9 ± 54.4	0.627
CrCl < 80 mL/min [no. (%)]	7 (30.4)	5 (29.4)	2 (33.3)	NS
Serum creatinine level (µmmol/L)	54.4 (39.7–82.6)	50.5 (36.5–86.3)	63 (55.4–132.2)	0.224
Primary Site of XDR-GNB infection [no. (%)]				0.025
Respiratory infection	17 (73.9)	15 (88.2)	2 (33.3)	
Bloodstream infection	1 (4.3)	0 (0)	1 (16.7)	
Other infections^b^	5 (21.7)	2 (11.8)	3 (50.0)	
Causative XDR-GNB [no. (%)]				0.148
*Acinetobacter* baumannii	12 (50.0)	11 (61.1)	1 (16.7)	
*Pseudomonas aeruginosa*	7 (29.2)	4 (22.2)	3 (50.0)	
*Klebsiella pneumoniae*	5 (20.8)	3 (16.7)	2 (33.3)	
Concomitant antibiotics [no. (%)]				0.876
Tigecycline	15 (65.2)	11 (64.7)	4 (66.7)	
Ceftazidime avibactam	1 (4.4)	1 (5.9)	0 (0)	
Carbapenems	4 (17.4)	3 (17.6)	1 (16.7)	
Others^c^	3 (13.0)	2 (11.8)	1 (16.7)	
PMB treatment				
Loading dose (mg/Kg/d)	2.0 ± 0.67	1.96 ± 0.77	2.10 ± 0.22	0.687
Daily maintain dose (mg)	180.76 ± 22.91	173.97 ± 23.10	200	0.013
Daily maintain dose/weight (mg/Kg/d)	2.63 ± 0.40	2.70 ± 0.34	2.41 ± 0.50	0.123
Daily maintaining dose ≥ 200 mg	12 (52.2)	6 (35.3)	6 (100)	0.014

All data are exhibited as number (%), mean ± standard deviation, or median (interquartile range). Others ^a^ include trauma, neurologic disease, gastrointestinal disease, connective tissue disease, shock, and cardiac failure. Other infections ^b^ include intra-abdominal infection and urogenital tract infection. Others ^c^ include cefoperazone-sulbactam, piperacillin/tazobactam, quinolones, and aminoglycosides. TBW, total body weight; CrCl, creatinine clearance; PMB, polymyxin B; XDR-GNB, extensively drug-resistant Gram-negative bacteria; NS, no significant.

The most common primary infection site of XDR-GNB was the respiratory tract in the elderly patients, and the intra-abdominal and urogenital tracts in the young patients, respectively. There was a total of 24 strains of XDR-GNB isolated from the infected specimens, of which the most common was *A. baumannii*. The isolated XDR-GNB strains were all sensitive to PMB with MICs ≤ 2 mg/L, of which 91.7% (22/24) of bacteria with MICs ≤ 0.5 mg/L, one bacteria with MIC = 1 mg/L and one bacteria with MIC = 2 mg/L. The most frequent causative XDR-GNB was *A. baumannii* in the elderly patients and *P. aeruginosa* in the young patients, respectively. The total daily maintenance doses of PMB and the proportions of patients with a total daily maintenance dose of PMB of more than 200 mg were higher in the young patients than in the elderly patients due to the higher TBW in the young patients (Table 1). However, the daily maintenance doses per TBW were similar between the two groups (Table 1).

### 3.2 The individual PK assessments of PMB

Overall, the AUC_ss, 0–24 h_ (range 11.30–151.28 mg·h/L) and the C_ss, avg_ (range 0.94–9.14 mg/L) were variable in the critically ill patients and only less than half of the critically ill patients could achieve the target AUC_ss, 0–24 h_ (50–100 mg·h/L) and the target C_ss, avg_ (2–4 mg/L) when prescribed with the daily dose calculated by the TBW as recommended by the current guideline listed in Table 2.

**TABLE 2:** Drug exposures and other individual pharmacokinetics parameters of polymyxin B in the elderly and young critically ill patients.

	All patients (*n* = 23)	Elderly group (*n* = 17)	Young group (n = 6)	*p*-value
AUC_ss,__0–12 h_ (mg·h/L)	50.26 ± 26.97	51.81 ± 25.27	45.86 ± 33.56	0.703
AUC_ss, 0–24 h_ (mg·h/L)	71.87 ± 41.79	76.24 ± 41.75	59.47 ± 43.06	0.431
Normalized AUC_ss, 0–24 h_ (mg·h/L)	52.31 ± 28.65	55.23 ± 28.51	44.03 ± 30.00	0.423
C_max_ (mg/L)	9.41 ± 3.12	9.23 ± 2.84	9.89 ± 4.07	0.725
C_min_ (mg/L)	2.99 ± 1.99	3.11 ± 1.79	2.65 ± 2.64	0.704
C_ss, avg_ (mg/L)	4.20 ± 2.23	4.32 ± 2.11	3.88 ± 2.75	0.730
T_max_ (h)	0.91 ± 0.49	0.941 ± 0.56	0.83 ± 0.26	0.536
Half-time time (h)	10.0 ± 5.07	11.21 ± 5.39	6.56 ± 1.16	0.003
Clearance (L/h)	2.49 ± 1.65	2.37 ± 1.76	2.85 ± 1.38	0.511
Clearance per TBW (L/h/kg)	0.036 ± 0.022	0.036 ± 0.024	0.037 ± 0.017	0.905
Volume of distribution (L)	24.68 ± 11.70	25.50 ± 12.94	22.33 ± 7.60	0.483
Volume of distribution per TBW (L/kg)	0.37 ± 0.17	0.40 ± 0.19	0.29 ± 0.09	0.085
C_ss, avg_ [no. (%)]				0.577
C_ss, avg_ < 2 mg/L	3 (13.0)	2 (11.8)	1 (16.7)	
C_ss, avg_ 2–4 mg/L	10 (43.5)	7 (41.2)	3 (50.0)	
C_ss, avg_ > 4 mg/L	10 (43.5)	8 (47.1)	2 (33.3)	
AUC_ss, 0–24 h_ [no. (%)]				0.265
AUC _0–24 h_ < 50 mg·h/L	8 (34.8)	5 (29.4)	3 (50.0)	
AUC _0–24 h_ 50–100 mg·h/L	7 (30.4)	5 (29.4)	2 (33.3)	
AUC _0–24 h_ > 100 mg·h/L	8 (34.8)	7 (41.2)	1 (16.7)	

All data are exhibited as number (%), mean ± standard deviation. AUCss, 0–24 h: the area under the concentration-time curve over 24 h at steady state; Css, avg: the average steady-state plasma concentration.

Neither the AUC_ss, 0–24 h_ nor the normalized AUC_ss, 0–24 h_ calculated by adjusting AUC to a daily dose of 1 mg/kg of TBW was significantly different between the elderly and young patients (Table 2). There were also no significant differences between the two groups in the proportions of patients with AUC_ss, 0–24 h_ less than 50 mg·h/L, patients with AUC_ss, 0–24 h_ ranging from 50 to 100 mg·h/L, and patients with AUC_ss, 0–24 h_ more than 100 mg·h/L. The other individual PK parameters, the C_ss, avg_, the C_max,_ and the C_min_, T_max_ were all comparable between the two groups (Table 2). Moreover, the proportions of patients with C_ss, avg_ less than 2 mg/L, patients with C_ss, avg_ ranging from 2 to 4 mg/L, and patients with C_ss, avg_ more than 4 mg/L were similar between the elderly and young patients. In addition, the clearance (L/h), clearance per TBW (L/h/Kg), volume of distribution (L), and volume of distribution per TBW (L/Kg) were comparable between the two groups (Table 2). However, the half-life time of elderly patients was significantly longer than the young patients (11.21 h vs. 6.56 h, respectively, *p* = 0.003) (Table 2), indicating that PMB metabolism or clearance was delayed in elderly critically ill patients.

### 3.3 Potential covariates of PMB PK in critically ill patients

Considering that the elderly patients had lower TBW than young patients in this study and body weight may affect the clearance and volume distribution of a drug, we further explored the potential covariates affecting PMB PK in all the critically ill patients. We did not find any statistically significant effects of age, SOFA score, total serum protein, albumin, serum creatinine, CrCl, fibrinogen, INR, and total bilirubin on PMB clearance and volume of distribution. Furthermore, there was no significant correlation of SOFA score, total serum protein, albumin, serum creatinine, CrCl, fibrinogen, INR, and total bilirubin with PMB Half-life time. However, age was moderately positively correlated with half-life time (*r* = 0.415, *p* = 0.049). In addition, TBW was positively correlated with clearance (*r* = 0.527, *p* = 0.010) and negatively correlated with half-life time (*r* = −0.489, *p* = 0.018). Next, we tried to elucidate the potential factors affecting the plasma concentration of PMB (AUC_ss, 0–24 h_, C_max_, C_min_, and C_ss, avg_). Unexpectedly, TBW was the only covariate with significant difference, which was negatively correlated with AUC_ss, 0–24 h_ (*r* = −0.414, *p* = 0.049), negatively correlated with C_min_ (*r* = −0.436, *p* = 0.038), and negatively correlated with C_ss, avg_ (*r* = −0.430, *p* = 0.041) but not correlated with C_max_.

### 3.4 Factors associated with PMB overdose

**FIGURE 2:**
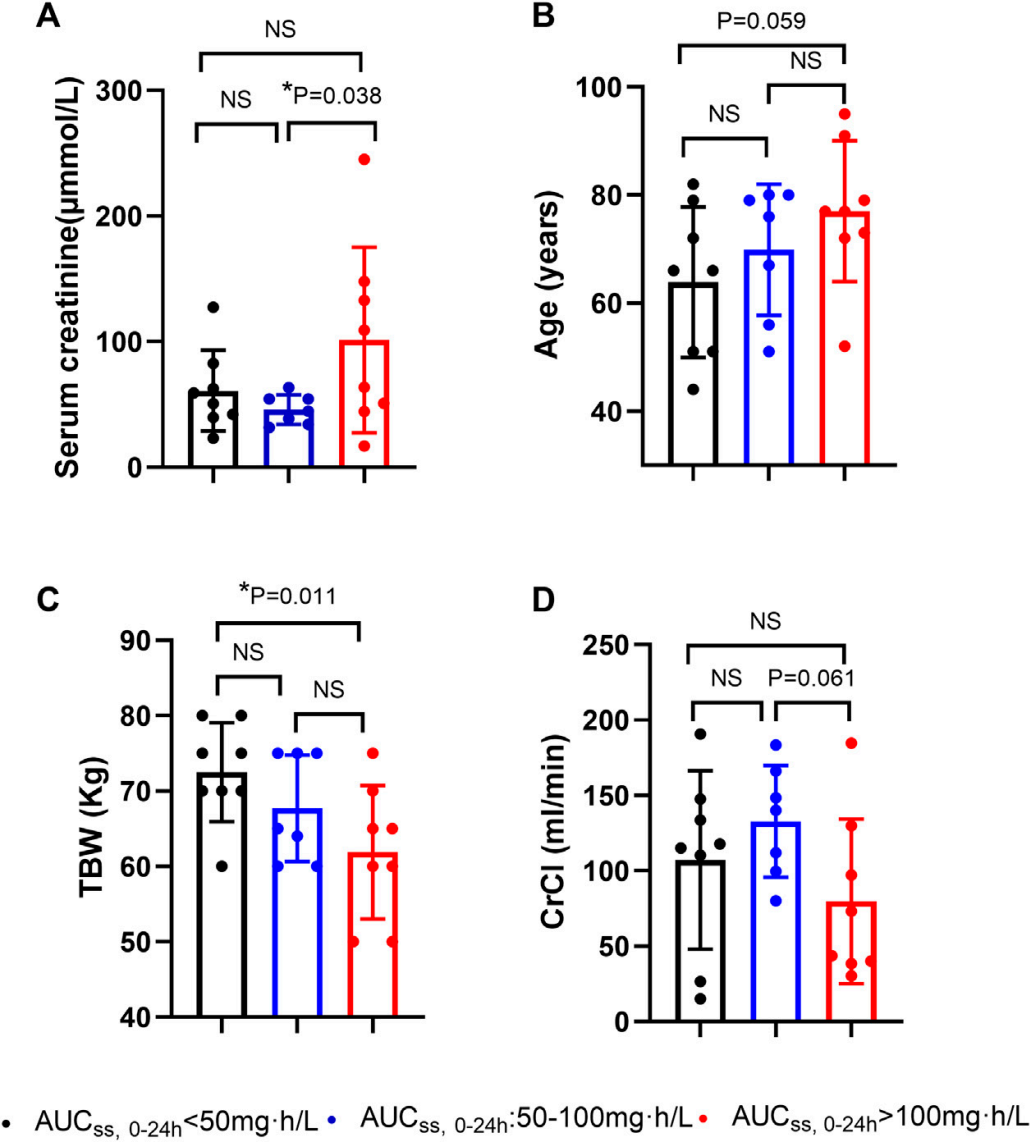
Potential factors of polymyxin B overdose in critically ill patients. **(A)** serum creatinine levels (µmmol/L); **(B)** age (years); **(C)** total body weight (TBW) (Kg); **(D)** CrCl (creatinine clearance) (ml/min). AUC_ss, 0–24 h_: the area under the concentration-time curve over 24 h at steady state.

We then further compared the levels of the above-mentioned potential covariates among patients with AUC_ss, 0–24 h_ less than 50 mg·h/L, patients with AUC_ss,__0–24 h_ ranging from 50 to 100 mg·h/L, and patients with AUC_ss, 0–24 h_ more than 100 mg·h/L. The patients with AUC_ss, 0–24 h_ more than 100 mg·h/L had significantly higher serum creatinine levels and significantly lower TBW than the patients with AUC_ss, 0–24 h_ less than 50 mg·h/L and patients with AUC_ss, 0–24 h_ ranging from 50 to 100 mg·h/L (Figure 2). The patients with AUC_ss, 0–24 h_ more than 100 mg·h/L were older than patients with AUC_ss, 0–24 h_ less than 50 mg·h/L (*p* = 0.059) and had lower CrCl than patients with AUC_ss, 0–24 h_ ranging from 50 to 100 mg·h/L (*p* = 0.061) (Figure 2), but the *p* values did not reach statistical significance. These results indicated that the lower TBW and higher baseline serum creatinine levels were more likely to result in AUC_ss, 0–24 h_ of PMB more than 100 mg·h/L, which would increase the risk of PMB-induced AKI as reported previously (Wang et al., 2021a; Yang et al., 2022).

## 4 Discussion

This PK study has made a significant contribution to understanding how to optimize the use of PMB in clinical practice in critically ill patients. To our knowledge, this is the first study to date comparing PMB exposure and other individual PK parameters in critically ill patients of different ages given the standard dosing of PMB. Age was not shown to be correlated with clearance and volume of distribution of PMB in critically ill patients in this study, consistent with many previously published studies (Sandri et al., 2013; Avedissian et al., 2018; Manchandani et al., 2018; Wang et al., 2020; Wang et al., 2021a; Yu et al., 2021; Surovoy et al., 2022; Yang et al., 2022). However, we did find that the elderly patients had longer half-life time than the young critically ill patients and age was positively correlated with half-life time of PMB. Half-life time is an important factor influencing the AUC_ss, 0–24 h_ and was shown to be positively correlated with AUC_ss, 0–24 h_ (*r* = 0.688, *p* < 0.001) in this study. Although the mean AUC_ss, 0–24 h_ was not significantly different between the elderly and the young patients, the age of the patients with AUC_ss, 0–24 h_ more than 100 mg·h/L was higher than that of patients with AUC_ss, 0–24 h_ less than 50 mg·h/L, with marginal difference. These results indicate that elderly patients may have more delayed PMB clearance and metabolism than young patients and trend toward PMB overdose given the standard dosage.

Factors influencing the distribution, metabolism, and excretion of a drug can increase its half-life time. We speculate a few possible explanations for why elderly patients have longer half-life time than young patients. Firstly, in comparison with the young patients, the elderly patients had lower TBW, which was shown to be positively correlated with PMB clearance in this study. Consistently, three studies have demonstrated a significant positive correlation of TBW with clearance or volume of distribution of PMB (Sandri et al., 2013; Miglis et al., 2018; Crass et al., 2021). Our study also found TBW was negatively correlated with AUC_ss, 0–24 h_ while the AUC is dependent on the dose and clearance of a drug, indicating that the TBW affects the AUC mainly through influencing PMB clearance. For example, the prescribed PMB dose was calculated by TBW in this study. If a patient has low TBW, the given dose of PMB would decrease, and PMB clearance would decrease while the AUC_ss, 0–24 h_ would increase, according to our study. Only when a decrease in clearance is greater than the decrease in the given dose, an increase in AUC could be observed. However, the relationship between TBW and clearance and volume of distribution of PMB was not certain. In contrast to our study, many PK studies did not find TBW as a covariate of the PMB clearance and volume of distribution (Avedissian et al., 2018; Kubin et al., 2018; Manchandani et al., 2018; Wang et al., 2020; Wang et al., 2021a; Wang et al., 2021b; Li et al., 2021; Yu et al., 2021), and weight-based dosage regimens are being challenged based on these findings (Liu et al., 2023). Therefore, such discrepancy could be caused by several factors, Such as the difference in sample size, with a range of 9–70 (Sandri et al., 2013; Avedissian et al., 2018; Kubin et al., 2018; Manchandani et al., 2018; Miglis et al., 2018; Wang et al., 2020; Wang et al., 2021a; Wang et al., 2021b; Crass et al., 2021; Li et al., 2021; Yu et al., 2021), different compartment models, and different dosing regimens used in various studies. For instance, PMB was administered with loading doses first or directly administered with maintenance doses ranging from 0.34 to 3.45 mg/kg/day (Sandri et al., 2013; Avedissian et al., 2018; Kubin et al., 2018; Manchandani et al., 2018; Miglis et al., 2018; Wang et al., 2020; Wang et al., 2021a; Wang et al., 2021b; Crass et al., 2021; Yu et al., 2021) or with the fixed dose of 40–100 mg twice a day in these studies (Wang et al., 2020; Wang et al., 2021a; Crass et al., 2021; Li et al., 2021; Chen et al., 2022). The study population was also different, including critically ill patients and patients with cystic fibrosis, renal transplantation, extracorporeal membrane oxygenation, or different renal function (Avedissian et al., 2018; Wang et al., 2021a; Crass et al., 2021; Li et al., 2021; Yu et al., 2021; Surovoy et al., 2022), and some studies did not mention the disease severity of the enrolled patients (Avedissian et al., 2018; Kubin et al., 2018; Manchandani et al., 2018; Miglis et al., 2018; Wang et al., 2020; Wang et al., 2021a; Crass et al., 2021; Li et al., 2021). The pharmacokinetics of antibiotics are more profoundly altered in critically ill patients than in general patients and healthy volunteers due to many pathophysiological changes during acute critical illness such as capillary leak, edema, reduced serum albumin concentration, altered renal blood flow, etc. (Gonçalves-Pereira et al., 2021).

Secondly, the elderly patients had higher Charlson comorbidity scores than the young patients in this study. This means the elderly patients had more co-morbidities and more medications for their co-morbidities and tended to have a reduced renal glomerular filtration rate and hepatic metabolism. However, the metabolism and excretion of PMB remain unclear. Generally, PMB is mainly eliminated by non-renal pathways as a very low urine recovery of PMB was found after intravenous injection (Zavascki et al., 2008; Sandri et al., 2013). Hepatic biliary excretion could be one of the non-renal routes of PMB elimination because all four major PMB components were detected in bile over 4 h after intravenous injection (Manchandani et al., 2015). Due to the unclear metabolism routes, we cannot identify which type of medications or which underlying co-morbidities could interfere with the metabolism and excretion of PMB.

The optimal PMB dosing regimen is controversial currently. Determination of clinical variables influencing PMB exposure and the enhancement of recognition of healthcare professionals worldwide through education is critical to optimize dosing regimens to improve patient outcomes. The surprising findings in this study are that the lower total body weight and higher baseline serum creatinine levels were the significant clinical factors associated with PMB overdose. Also, total body weight was the only variable with a significant correlation with PMB plasma concentration as shown in this study. The relationship between body weight and PMB exposure is an unexpected result that has not been reported previously and deserves further study with a large population for verification. As for higher baseline serum creatinine, in contrast with the current guideline, which recommends there is no need to adjust PMB dosage in patients with renal insufficiency (Tsuji et al., 2019), our result indicates the adjustment of PMB dosing is needed in patients with higher serum creatinine levels to minimize the risk of nephrotoxicity. Theoretically, the pathophysiological changes in renal impairment or age-related physiological changes such as decreased glomerular filtration rate and tubular absorption dysfunction could decrease PMB clearance and result in higher PMB exposure (Avedissian et al., 2019; Nie et al., 2022). Similarly to our study, Wang et al. found that the mean AUC_ss, 24 h_ of PMB in their renal insufficiency group was higher than in their normal renal function group, with marginal significance (Wang et al., 2021a). Moreover, some clinical studies have demonstrated that the patients with PMB-associated AKI had higher AUC_ss, 0–24 h_ (Xu et al., 2022; Yang et al., 2022), higher baseline serum creatinine levels, or lower baseline glomerular filtration rates compared to patients without PMB-associated nephrotoxicity (John et al., 2017; Xi et al., 2022; Xu et al., 2022). These results support the speculation that patients with renal dysfunction are more likely to develop higher PMB exposure and that the dosing regimen of PMB should be adjusted in patients with renal dysfunction. However, the relationship between renal function and PMB exposure is still under debate. Another study did not find that AUC_ss, 0–24 h_ or normalized AUC_ss, 0–24 h_ of PMB was significantly different between the patients with normal renal function and patients with renal insufficiency (Thamlikitkul et al., 2016).

The relationship between renal function and PMB clearance is also still under debate. Some population PK studies have demonstrated a significant positive correlation of CrCl with PMB clearance (Avedissian et al., 2018; Manchandani et al., 2018; Wang et al., 2020; Wang et al., 2021a; Li et al., 2021; Yu et al., 2021), which was not confirmed in other studies (Sandri et al., 2013; Miglis et al., 2018; Wang et al., 2021b; Crass et al., 2021; Surovoy et al., 2022; Wang et al., 2022; Liang et al., 2023). It is interesting to note that CrCl, but not other renal function markers such as serum creatinine levels, has been proven to be associated with PMB clearance in some studies (Avedissian et al., 2018; Manchandani et al., 2018; Wang et al., 2020; Wang et al., 2021a; Li et al., 2021; Yu et al., 2021). The CrCl was estimated using the Cockcroft-Gault formula, which is determined by age, TBW, sex, and serum creatinine levels in all the current PMB PK studies; however, the Cockcroft-Gault formula has been shown to be highly inaccurate for the assessment of renal function in critically ill patients (Bragadottir et al., 2013). Age and especially TBW were the possible covariates affecting PMB clearance shown by our group and other studies (Sandri et al., 2013; Miglis et al., 2018; Crass et al., 2021; Liang et al., 2023). Thus, it is possible that the significant correlation of CrCl with PMB clearance is influenced by age or body weight. Therefore, we cannot draw the conclusion that renal function is correlated with PMB clearance based on the findings of significant correlations of CrCl and PMB clearance. Furthermore, in the two studies about PMB exposure in patients with different renal function (Thamlikitkul et al., 2016; Wang et al., 2021a), renal insufficiency was diagnosed by estimating CrCl<80 mL/min using the Cockcroft-Gault formula. Whether the studied population was general patients or critically ill patients, and the proportion of critically ill patients in the enrolled patients were not mentioned in these two studies (Thamlikitkul et al., 2016; Wang et al., 2021b). Therefore, further studies aiming to explore the relationship between renal function and PMB clearance or PMB exposure should evaluate renal function using more accurate biochemical markers and match potential covariates including body weight, age, and study population.

Several limitations to our study must be considered. First, the small sample size. A larger sample size would enhance the robustness of our findings. Second, critically ill patients are highly heterogeneous and have more factors that may influence drug metabolism, distribution, and clearance compared with general patients. As such, it is unknown whether the results of this study can be generalized to the general patient population. However, critically ill patients are more vulnerable to XDR-GNB infection and develop severe infections with multiple organ dysfunctions more often than general patients. Therefore, critically ill patients are the population most worthy of study. Third, the difference in the association of drug exposure with clinical outcomes between the elderly and young critically ill patients was not compared in this study because the sample sizes in the two groups were too small to reach any definite conclusions.

In conclusion, PMB exposure at steady state was comparable between the elderly and the young critically ill patients. Elderly patients may have more delayed PMB clearance and metabolism than young patients. TBW was the only covariate affecting PMB clearance. High baseline serum creatinine levels and low TBW were associated with PMB overdose, indicating that the PMB dosing regimen needs to be adjusted in these patients to minimize the risk of nephrotoxicity. Further clinical studies with large sample sizes are urgently needed to investigate PMB PK in critically ill patients with different renal functions assessed using more accurate biochemical markers, different ages, and various body weights.

## Data Availability

The original contributions presented in the study are included in the article/supplementary material, further inquiries can be directed to the corresponding authors.
